# Electron-acoustic solitary potential in nonextensive streaming plasma

**DOI:** 10.1038/s41598-022-19206-4

**Published:** 2022-09-07

**Authors:** Khalid Khan, Obaid Algahtani, Muhammad Irfan, Amir Ali

**Affiliations:** 1 Department of Mathematics, University of Malakand, Dir (L), Khyber Pakhtunkhwa Pakistan; 2 Department of Mathematics, College of Sciences, King Saud University, P. O. Box 2455, Riyadh, 11451 Saudi Arabia; 3 Department of Physics, University of Malakand, Dir (L), Khyber Pakhtunkhwa Pakistan

**Keywords:** Physics, Applied mathematics

## Abstract

The linear/nonlinear propagation characteristics of electron-acoustic (EA) solitons are examined in an electron-ion (EI) plasma that contains negative superthermal (dynamical) electrons as well as positively charged ions. By employing the magnetic hydrodynamic (MHD) equations and with the aid of the reductive perturbation technique, a Korteweg-de-Vries (KdV) equation is deduced. The latter admits soliton solution suffering from the superthermal electrons and the streaming flow. The utility of the modified double Laplace decomposition method (MDLDM) leads to approximate wave solutions associated with higher-order perturbation. By imposing finite perturbation on the stationary solution, and with the aid of MDLDM, we have deduced series solution for the electron-acoustic excitations. The latter admits instability and subsequent deformation of the wave profile and can’t be noticed in the KdV theory. Numerical analysis reveals that thermal correction due to superthermal electrons reduces the dimensionless phase speed $(\bar{U}_{ph})$ for EA wave. Moreover, a random motion spread out the dynamical electron fluid and therefore, gives rise to $\bar{U}_{ph}$. A degree enhancement in temperature of superthermal (dynamical) electrons tappers of (increase) the wave steeping and the wave dispersion, enhancing (reducing) the pulse amplitude and the spatial extension of the EA solitons. Interestingly, the approximate wave solution suffers oscillation that grows in time. Our results are important for understanding the coherent EA excitation, associated with the streaming effect of electrons in the EI plasma being relevant to the earth’s magnetosphere, the ionosphere, the laboratory facilities, etc.

## Introduction

The reduction in binary interactions of the plasma components reduces the particles correlations that restore the ionized matter to non-extensive state. The latter has relevance to ionosphere^1^, magnetosphere^2^, solar winds^3^, laboratory plasma^4^, etc. Intriguingly, Maxwell’s statistics fail to describe the dynamics of particles in non-extensive plasmas. Vasyliunas^5^ introduced the distribution function that extended Lurentzian/kappa accounts correctly for the superthermal plasmas compositions. Importantly, a long tail associated with the Lorentzian/kappa particle distribution function shows deviation from the non-thermal thermodynamic equilibrium. Plasmas with low density and/or high-temperature^6^ have fewer binary collisions and correlation effects among components, and they can become non-thermal. In such plasmas, the statistical distribution of particles changes dramatically, rendering the traditional Maxwell-Gibbs statistics useless. The kappa or extended Lorentzian distribution function was initially developed by Vasyliunas^7^ to characterize the superthermal composition of the collisionless plasma in the magnetosphere. The extended Lorentzian function has a long-tailed particles distribution function, which deviates considerably from the thermodynamic equilibrium. Furthermore, when holds, the superthermal index ($\kappa _e\rightarrow \infty$) associated with non-thermal constituents restores a Maxwellian plasma state. It’s worth noting that superthermal particle states have been seen both in space and in laboratories. The reported thermal and superthermal velocity spectra for space plasmas^8–10^ match well with the Lorentzian distribution function. The electron fluid in laser-induced plasma^11^ achieves a nonequilibrium condition within the typical period, thus the kappa distribution function is suitable. The dispersion and damping rates measured for electron-acoustic waves (EAWs) in laboratory plasma^12^ precisely match the calculated superthermal index $\kappa _e$ range of 3−4, validating the Lorentzian distribution function for hot electrons. Sultana et al.^13^ studied the nonlinear development of ions acoustic (IA) excitations in plasma with kappa distributed electrons and discovered that superthermal electrons permit smaller shocks with greater amplitude. The kappa dispersed ions in magneto-dusty electron depletion plasma ignite the negative polarity oblique dust-acoustic isolated potentials studied by Shahmansouri and Alinejad in^14^. The Lorentzian plasma approximation may also be used to wave dynamics and related instabilities in the interstellar medium^15^, solar wind^16^, ionosphere^17^, auroral zone^18^, and other areas.

Fried and Gould^19^ first put forward an idea for excitation of the electron-acoustic (EA) mode. They have pointed out that the EA potentials suffer a Landau’s damping effect that decreases with the increase of wave number. In the later investigations^20^ revealed weak damping of the EA in plasma, that contains both high and low-temperature electrons. Such plasma conditions had already been observed in diverse contexts^21^. Iwamoto^22^ has examined the evolution of electron-acoustic wave as well as the high frequency Langmuir mode in nonrelativistic electron-positron plasma. It has been shown therein^22^ that the low frequency EA excitation Landau damped with a relatively larger growth rate in comparison of the Langmuir wave. Saberian and Esfandyari-Kalejahi^23^ have investigated the propagation characteristic of Langmuir excitations in nonextensive electron-positron plasma. They have pointed out that super thermal electron/positron give rise to damping/growth of the Langmuir waves. Saberian et al.^24^ investigated the high frequency Langmuir waves in nonextensive electron-positron plasma. It has been shown that, superthermal electron/positron cause damping/growth of the longitudinal waves. Importantly, a broadband electrostatic noise (BEN) observed at the Earth’s magnetosphere^25^ as well as at the auroral zone^26^, have confirmed the evolution of EA perturbations. The BEN is thought to be created by EA solitons traveling fast the observing spacecraft. Indeed, EAW solitons have been seen in wave activity in the auroral area and the geomagnetic tail using FAST satellite data^27^. Mace and Hellberg^28^ studied the impact of a magnetic field on such electron acoustic solitons using a Korteweg-de Vries-Zakharov-Kuznetsov (KdV-ZK) model, and addressed its significance to the challenge of BEN interpretation.

We investigate the amplitude modulation of electron acoustic waves (EAWs) using the reductive perturbation approach^29^ and a one-dimensional model of a plasma comprised of a cold electron fluid and hot electrons obeying the kappa type of distribution. Using the traditional reductive perturbation approach and the nonlinear field equations of such a plasma we obtained a non-linear KdV equation for our modulation. When comparing the result of plasma with vortex distribution to a solitary wave solution with advancing amplitude wave to the evolution equation, it is discovered that the amplitude wave takes the form of a solitary wave.

Nonlinear complex physical processes in Plasma physics are well recognised to be related to nonlinear partial differential equations (NLPDEs). In general, obtaining localized solutions for NLPDEs such as the non-linear Korteweg-de-Vries (KdV) is a difficult task. As a result, some recently improved approximate solutions have been developed to overcome on this issue, for example to solve a NLPDEs, researchers used the analytical methods like homotopy perturbation approach^30,31^, sine-cosine method^32^ and modified simple equation method^33^. To explore the nonlinear differential equation, a powerful combination of an auxiliary parameter approach combining adomain polynomials and Laplace transformation^34^. The purpose of this work is to look at how to solve the non-linear Korteweg-de-Vries (KdV) equation by using the modified double Laplace decomposition method (MDLDM)^35,36^. The proposed method is the combination of a double Laplace transform and Adomian decomposition methods. This method is an authentic tool for solving the approximate solution for a non-linear problems appear in plasma physics. For example, the acoustic waves generated in the ionosphere may have associated with a very high amplitude due to the higher magnetic field. Modified double Laplace decomposition method (MDLDM) gives a series solution contain the higher order time dependent terms to the non-linear KdV, which may helpful to reduce the high amplitudes of the EAWs.

By employing the Vlaso-Poisson simulations, Valentini et al.^37^ have described evolution of the undamped electron-acoustic waves as well as the Langmuir excitations termed as the corner modes. They have also illustrated the regime for thumb curve where theses modes coexist. Similarly, the results for sheath formation in electron-ion plasma with superthermal electrons are summarised, and obtained generalized Bohm criterion^24^. It should be noted that, our analysis relies on weakly nonlinear and weakly dispersive EA solitary potentials in magnetoplasma with streaming electrons. By imposing the modified double laplace decomposition (MDLD) method, we have derived distinct numerical solution for EA potential superimposed by finite perturbations. The latter admits instability and subsequent deformation against finite perturbations. These results are not elaborated elsewhere.

The rest of the paper is organized as follows: In “Governing equations and model” section, we consider the fluid equations (MHD) in the component form. In “Linear stability/instability analysis” section, the linear behaviour of the MHD equation is discussed for stability analysis of the shear flow of EA plasma. “Non-linear wave analysis” section consists of non-linear analysis of the model. The KdV equation is derived for localized solutions as well as for superthermal plasma using a reductive perturbation technique. In “Modified double Laplace decomposition method (MDLDM)” section, we discuss some definitions of the proposed method and apply the technique to a general non-linear differential equation. In “Applications of MDLDM” section, we apply the proposed method to obtain an approximate solution. The effect of different parameters on the phase speed $U_{ph}$, the nonlinearity coefficient *A*, and the dispersive coefficient *B* is discussed in the same section. The results and discussion are presented in “Results and discussions” section. The conclusion derived from the paper is given in “Conclusion” section.

## Governing equations and model

Here, we study the propagation characteristics for the electron-acoustic (EA)solitons in a nonnegative-ion (EI) plasma that comprises dynamical electrons, superthermal hot electrons, and stationary ions. The EI plasma is assumed to be immersed in a uniform magnetic field ($B^{(0)}\hat{Z}$) in *Z*-direction. It is assumed that the electrons supper a constant shear flow $(U^{(0)}=a\hat{Z})$ in the *Z*-axis, where *a* stands for the magnitude of the speed. Importantly, at electron dynamical scale the phase speed for EA wave is much larger as compared to the thermal speed of electrons, i.e $\omega /K<<U_{th}$ condition holds. Here $U_{Th}(=\sqrt{K_BT_h/m_e})$ represent the thermal speed, with $K_B$ the Boltzmann constant and $T_h$ is the temperature of the hot electrons. The nonlinear evaluation of EA mode is governed by the following fluids equations

1 $$\begin{aligned}{}&\frac{\partial N_{c}}{\partial T}+\nabla \left( N_{c}U_{c}\right) =0, \end{aligned}$$

2 $$\begin{aligned}{}&\quad m_{e}N_{c}\left( \frac{\partial U_{c}}{\partial T}+U_{c}\cdot \nabla U_c\right) =eN_{c}\left( E+\frac{U_{c}}{C}\times B^{(0)}\right) -\nabla P_c, \end{aligned}$$

and

3 $$\begin{aligned} \nabla ^2 \Phi =4\pi e\left( N_c+N_h-N_{i0}\right) , \end{aligned}$$

where $N_c$ ,$U_{c}$ and $P_c$ designated the number densities, speed and pressure respectively for the cold electrons. Moreover, $\Phi$ is the electrostatic potential, $e(m_e)$ is the electronic charge (mass) and $N_h(N_i)$ represents the number densities for hot electrons (ions).

The hot electrons can be taken as inertialess for much large energy and therefore described by the following kappa distribution function (Baluku and Helberg^38^)

4 $$\begin{aligned} N_h=N_{h}^{(0)}\left\{ 1-\frac{e\Phi }{K_BT_h(\kappa _e-\frac{3}{2})}\right\} ^{-\kappa _e+\frac{1}{2}} , \end{aligned}$$

where the index ($\kappa _e$) accounts for the superthermal electrons. In the presence of magnetic field, the electrons may experiences the drift motion as

5 $$\begin{aligned} \begin{aligned} U_{c\perp }&=U_{E}+U_{p}+U_{D}, \end{aligned} \end{aligned}$$

where $U_{E}\big \{=C\left( E_{\perp }\times \hat{Z}\right) /B^{(0)}\big \}$, are the electric drift, $U_{p}\{=-(\left( \partial _t+ U_{c}\cdot \nabla \right) U_{c\perp }\times \hat{Z})/\Omega _c\}$ polarization drift and $U_{D}\{=(-U_{Tc}^2\nabla _{\perp }\cdot N_c\times \hat{Z})/\Omega _{c}N_{c}\}$ are diamagnetic drift of the electron. The velocity component associated with the dynamical electrons turns out to be

6 $$\begin{aligned} U_{c}=U^{(0)}+U_{cX}+U_{cY}+U_{cZ}+U_{c\perp }. \end{aligned}$$

By using Eqs. (4–6) and after some algebraic manipulation, one can reduce Eq. (1) as

7 $$\begin{aligned} \begin{aligned}{}&\frac{\partial N_{c}}{\partial T}+\left( U_{cX}\frac{\partial }{\partial X}+U_{cY}\frac{\partial }{\partial Y}+U_{cZ}\frac{\partial }{\partial Z}\right) N_{c}+N_c\left( \frac{\partial U_{cX}}{\partial X}+\frac{\partial U_{cY}}{\partial Y}+\frac{\partial U_{cZ}}{\partial Z}\right) \\ {}&+\frac{CN_c}{B^{(0)}\Omega _{c}}\frac{\partial }{\partial T}\left( \frac{\partial ^2}{\partial X^2}+\frac{\partial ^2}{\partial Y^2}\right) \Phi -\frac{U_{T_c}^2N_c}{\Omega _{c}}\left( \frac{\partial N_c}{\partial X}\frac{\partial }{\partial Y}-\frac{\partial N_c}{\partial Y}\frac{\partial }{\partial X}\right) \left( \frac{N_c}{N_c^{(0)}}\right) ^2=0 ,\end{aligned} \end{aligned}$$

where $\Omega _{c}(=eB^{(0)}/Cm_e)$, $U_{T_c}(=\sqrt{K_BT_c/m_e})$ designate the electron gyro frequency and the acoustic speed respectively with *C* is speed of light. The components of Eq. (2) in *X*, *Y* and *Z*-direction are

8 $$\begin{aligned}{}&\frac{\partial U_{cX}}{\partial T}+U_{cX}\frac{\partial U_{cX}}{\partial X}-\frac{e}{m_e}\frac{\partial \Phi }{\partial X}-\Omega _cU_{cY}+\frac{3U_{T_c}^2N_c}{(N_c^{(0)})^2}\frac{\partial N_{c}}{\partial X}= 0, \end{aligned}$$

9 $$\begin{aligned}{}&\quad \frac{\partial U_{cY}}{\partial T}+U_{cY}\frac{\partial U_{cY}}{\partial Y}-\frac{e}{m_e}\frac{\partial \Phi }{\partial Y}-\Omega _cU_{cX}+\frac{3U_{T_c}^2N_c}{(N_c^{(0)})^2}\frac{\partial N_{c}}{\partial Y}= 0, \end{aligned}$$

10 $$\begin{aligned}{}&\quad \frac{\partial U_{cZ}}{\partial T}+U_{cZ}\frac{\partial U_{cZ}}{\partial Z}+\frac{U^{(0)}\partial U_{cZ}}{\partial Z}-\frac{e}{m_e}\frac{\partial \Phi }{\partial Z}-\Omega _cU_{cZ}+\frac{3U_{T_c}^2N_c}{(N_c^{(0)})^2}\frac{\partial N_{c}}{\partial Z}=0, \end{aligned}$$

Equation (3) can be expressed in the form as

11 $$\begin{aligned} \left( \frac{\partial ^2 }{\partial X^2}+\frac{\partial ^2 }{\partial Y^2}+\frac{\partial ^2 }{\partial Z^2}\right) \Phi =4\pi e\left( N_c+N_h-N_i^{(0)}\right) . \end{aligned}$$

Equations (7)–(11) describe the evolution of electron-acoustic excitations in non-extensive plasma that comprises of dynamical electrons as well as kappa distributed electrons and stationary ions.

## Linear stability/instability analysis

In order to examine stability/Instability conditions of a linear mode, we expand the relevant parameters in Eqs. (8)–(11) in the form exp$[i(K_xX+K_yY+K_zZ-\omega T)]$ up to first order. Thus after some algebraic manipulation, we obtain the following quartic equation

12 $$\begin{aligned} \begin{aligned} \left( C_0-\frac{CN_c^{(0)}K_\perp ^2}{B^{(0)}\Omega _c}\right) \omega ^4&+ \left( \frac{CN_c^{(0)}{U^{(0)}}K_\parallel K_\perp ^2}{B^{(0)}\Omega _c}-2C_0(U^{(0)})K_\parallel \right) \omega ^3\\&+\left( C_0(U^{(0)})^2K_\parallel ^2-C_0\Omega _c^2+\frac{eN_c^{(0)} K_\perp ^2}{m_e}-\frac{3C_0K_BT_cK_\perp ^2}{m_e}+D_0K_\parallel ^2+\frac{CN_c^{(0)}\Omega _c K_\perp ^2}{B^{(0)}}\right) \omega ^2\\ {}&+\bigg (2U^{(0)}C_0K_\parallel \Omega _c^2-\frac{e\,U^{(0)}N_c^{(0)}K_\parallel K_\perp ^2}{m_e}+\frac{3C_0K_BT_c\,U^{(0)}K_\parallel K_\perp ^2}{m_e}\\ {}&-\frac{CU^{(0)}N_c^{(0)}\Omega _cK_\parallel K_\perp ^2}{B^{(0)}}\bigg )\omega -C_0(U^{(0)})^2K_\perp ^2\Omega _c^2-D_0K_\parallel ^2\Omega _c^2=0, \end{aligned} \end{aligned}$$

**Figure 1:**
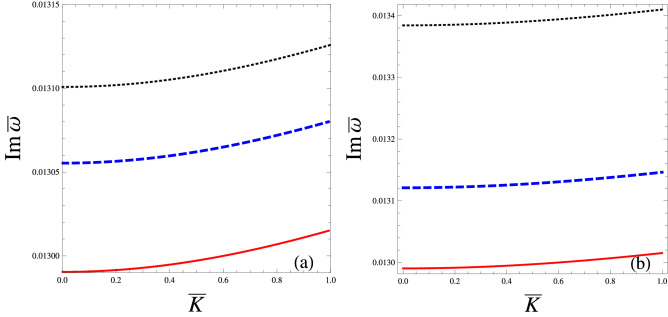
The dimensionless imaginary root $Im\bar{\omega }$ (where$\quad \bar{\omega }=\omega /\omega _0$ with $\omega _0=10^{12}\,\mathrm{rad}/\mathrm{s})$ in Eq. (12), is plotted against the dimensionless wavenumber $\bar{K}(=K/K_0$ with $K_0=10^{6}\,\mathrm{cm}^{-1}$) with variation in (**a**) streaming speed $U^{(0)}=10^3\,\mathrm{cm}\,\mathrm{s}^{-1}$ (solid curve), $2\times 10^3\,\mathrm{cm}\,\mathrm{s}^{-1}$ (dashed curve) and $3\times 10^3\,\mathrm{cm}\,\mathrm{s}^{-1}$ (dotted curve). The same is depicted versing $\bar{K}$ when (**b**) magnetic field $B^{(0)}$=$10^5G$ (solid curve), $2\times 10^5G$ (dashed curve) and $3\times 10^5G$ (dotted curve) when $T_c=10^3\hbox {K}(T_h=10^4K)$ and $\kappa _e=3$ .

where $$C_0\left( =(a_0-K^2)/4\pi e\right) $$,   $$D_0\left( =e/m_e-3K_BT_cA_0/m_eN_c^{(0)}\right) $$, with $$C_1\left( =(-\kappa _e+1/2)/(\kappa _e+3/2)\right) $$,

$a_0\left( =4\pi e^2N_h^{(0)}C_1/K_BT_h\right)$, $K_\perp ^2\left( =K_x^2+K_y^2\right)$,   $K_\parallel ^2\left( =K_z^2\right)$ and $K\left( =\sqrt{K_\parallel ^2+K_\perp ^2}\right)$. Notice that in the limit when $U^{(0)}=0$, the coefficients of $\omega ^3$ and $\omega$ vanish and therefor Eq. (12) reduces into a biquadratic equation as already obtained in^29^. Our interest is in the case when $U^{(0)}\ne 0$, the numerical solution of Eq. (12) reveals real as well as imaginary roots. The dimensionless imaginary root ($Im\,\bar{\omega }$) in Eq. (12), corresponds to instability growth rate, depicted versus dimensionless wavenumber ($\bar{K})$ in Fig. 1a with variations in streaming speed $U^{(0)}=10^3\,\mathrm{cm}\,\mathrm{s}^{-1}$ (solid curve), $2\times 10^3\,\mathrm{cm}\,\mathrm{s}^{-1}$(dashed blue curve) and $3\times 10^3\,\mathrm{cm}\,\mathrm{s}^{-1}$(dotted black curve). See the streaming effect of magnetoplasma rises the instability growth rate. The same is given in Fig. 1b versus $\bar{K}$ when the magnetic field $B^{(0)}=10^5G$ (solid curve), $2\times 10^5G$ (dashed curve) and $3\times 10^5G$(dotted curve). It reveals that the intensification in the $B^{(0)}$ favors instability of the linear EA waves.

## Non-linear wave analysis

For the nonlinear evaluation of electron-acoustic excitations in the nonextensive EI plasma, we use the reductive perturbation technique given by Washimi and Tanuili^39^. In this context, we chose the following stretching and the spatial-temporal variables as

13 $$\begin{aligned} \zeta =\epsilon ^{1/2}(K_xX+K_yY+K_zZ-U_{ph}T), \quad \,\,\,\,\tau =\epsilon ^{3/2}T, \end{aligned}$$

where $U_{ph}$ is phase velocity of the waves, $K_x$, $K_y$ and $K_z$ are the direction cosines of the wave vector along *X*, *Y* and *Z*-axis respectively.

The relevant plasma parameters are represented in the form as

14 $$\begin{aligned} \begin{pmatrix}U_{cZ}\\ N_{c}\\ \Phi \end{pmatrix} =\begin{pmatrix}0\\ N_{c}^{(0)}\\ 0\end{pmatrix}+\epsilon \begin{pmatrix} U_{cZ}^{(1)}\\ N_{c}^{(1)}\\ \Phi ^{(1)}\end{pmatrix}+\epsilon ^2 \begin{pmatrix} U_{cZ}^{(2)}\\ N_{c}^{(2)}\\ \Phi ^{(2)},\end{pmatrix}+\cdots , \end{aligned}$$

similarly, the transverse components of electrons speed can be expressed as

15 $$\begin{aligned} \begin{pmatrix}U_{cX}\\ U_{cY}\end{pmatrix} =\begin{pmatrix}0\\ 0\end{pmatrix}+\epsilon ^{\frac{3}{2}}\begin{pmatrix} U_{cX}^{(1)}\\ U_{cY}^{(1)}\end{pmatrix}+\epsilon ^2\begin{pmatrix} U_{cX}^{(2)}\\ U_{cY}^{(2)}\end{pmatrix}+\cdots , \end{aligned}$$

it should be noted that $\epsilon$ is a trivially very small dimensionless factor that calculates the energy of the dispersion and non-linearity. The occurrence of the magnetic field $B^{(0)}$ in the system leads to anisotropy, because of which the perpendicular components ($U_{cX}$ and $U_{cY}$) can be stated in an upper order of the parameter $\epsilon$ than the corresponding factors of velocity $U_{cZ}$. Therefore, the gyro-motion effects in the higher-order influence in the model. The transverse velocity components are extended to jump with $\epsilon ^{3/2}$, while the corresponding component of velocity starts with $\epsilon$. The components having $\epsilon ^{3/2}$ represent weak velocity perturbation as compared to the component having order $\epsilon$.

By using Eqs. (13–15) in Eqs. (7–11) we get lowest orders in $\epsilon$

16 $$\begin{aligned} U_{cY}^{(1)}&=  -\frac{CK_x}{B^{(0)}}\left( 1+\frac{3U_{Tc}^2}{U_{Th}^2} \frac{C_1N_{h}^{(0)}}{N_{c}^{(0)}}\right) \frac{\partial \Phi ^{(1)}}{\partial \zeta }, \end{aligned}$$

17 $$\begin{aligned} U_{cX}^{(1)}&=  -\frac{CK_y}{B^{(0)}}\left( 1+\frac{3U_{Tc}^2}{U_{Th}^2} \frac{C_1N_{h}^{(0)}}{N_{c}^{(0)}}\right) \frac{\partial \Phi ^{(1)}}{\partial \zeta }, \end{aligned}$$

18 $$\begin{aligned} U_{cZ}^{(1)}&=  \frac{C_1eN_{h}^{(0)}}{K_{B}T_h}\left( U^{(0)}-\frac{U_{ph}}{K_z}\right) \frac{\partial \Phi ^{(1)}}{\partial \zeta }, \end{aligned}$$

19 $$\begin{aligned} \frac{\partial }{\partial \zeta }U_{cZ}^{(1)}&=  \frac{U^{(0)}K_z}{U_{ph}}\frac{\partial U_{cZ}^{(1)}}{\partial \zeta }+\frac{U^{(0)}K_z}{U_{ph}}\frac{\partial U_{cZ}^{(1)}}{\partial \zeta }-\frac{eK_z}{U_{ph}m_e}\frac{\partial \Phi ^{(1)}}{\partial \zeta }+\frac{3U_{T_c}^2K_z}{U_{ph}N_{c}^{(0)}}\frac{\partial N_{c}^{(1)}}{\partial \zeta }, \end{aligned}$$

20 $$\begin{aligned} N_{h}^{(1)}&=  -\frac{C_1eN_h^{(0)}}{K_BT_h}\Phi ^{(1)}=N_{c}^{(1)}, \end{aligned}$$

where $C_1=(\kappa _e-1/2)/(\kappa _e-3/2)$. By solving Eqs. (15–19) we can acquire phase speed of the EAWs as

21 $$\begin{aligned} U_{ph}=\left( U^{(0)}+\sqrt{3C_3U_{T_c}^2m_e^2(N_{h}^{(0)})^2+eN_c^{(0)}}\right) K_z, \end{aligned}$$

where $C_3=C_1e N_{h}^{(0)}/K_BT_h$ stands for an expansion parameter. The expansion of the perturbation series beyond the first order of $\epsilon$ leads to the following perturbations

22 $$\begin{aligned} U_{cY}^{(2)}&=  \frac{m_eU_{ph}K_y}{e}\left( 1+\frac{3U_{Tc}^2}{U_{Th}^2}\frac{C_1N_{h}^{(0)}}{N_{c}^{(0)}}\right) \frac{\partial ^2\Phi ^{(1)}}{\partial \zeta ^2}, \end{aligned}$$

23 $$\begin{aligned} U_{cX}^{(2)}&=  \frac{m_eU_{ph}K_x}{e}\left( 1+\frac{3U_{Tc}^2}{U_{Th}^2}\frac{C_1N_{h}^{(0)}}{N_{c}^{(0)}}\right) \frac{\partial ^2\Phi ^{(1)}}{\partial \zeta ^2}, \end{aligned}$$

24 $$\begin{aligned} \frac{\partial ^2\Phi ^{(1)}}{\partial \zeta ^2}&=  4\pi e\left( N_{c}^{(2)}+\frac{C_1eN_{h}^{(0)}}{K_BT_h}\Phi ^{(2)}+C_2N_{h}^{(0)}\frac{e^2}{K_B^2T_h^2}(\Phi ^{(1)})^2\right) , \end{aligned}$$

where $C_2=-(-\kappa _e+\frac{1}{2})(-\kappa _e-\frac{1}{2})/2(\kappa _e-\frac{3}{2})$. The next higher orders in $\epsilon$ for momentum and continuity equations respectively are

25 $$\begin{aligned} U_{ph}\frac{\partial U_{cZ}^{(2)}}{\partial \zeta }&=2U^{(0)}K_z\frac{\partial U_{cZ}^{(2}}{\partial \zeta }+\left( U_{cZ}^{(1)}K_z\frac{\partial }{\partial \zeta }+\frac{\partial }{\partial \tau }\right) U_{cZ}^{(1)}\nonumber \\&\quad +\left( \frac{3U_{T_c}^2K_z}{N_{c}^{(0)}}\frac{\partial }{\partial \zeta }+\frac{3U_{T_c}^2K_zN_{c}^{(1)}}{(N_{c}^{(0)})^2}\frac{\partial }{\partial \zeta }\right) N_{c}^{(2)} -\frac{e}{m_e}K_z\frac{\partial \Phi ^{(2)}}{\partial \zeta }, \end{aligned}$$

26 $$\begin{aligned} U_{ph}\frac{\partial N_{c}^{(2)}}{\partial \zeta }&=2U^{(0)}K_z\frac{\partial N_{c}^{(2)} }{\partial \zeta }+N_{c}^{(0)}\left( K_y\frac{\partial U_{cY}^{(2)}}{\partial \zeta }+K_x\frac{\partial U_{cX}^{(2)}}{\partial \zeta }+K_z\frac{\partial U_{cZ}^{(2)}}{\partial \zeta }\right) \nonumber \\&\quad +K_z\left( N_{c}^{(1)}\frac{\partial U_{cZ}^{(1)}}{\partial \zeta }+U_{cZ}^{(1)}\frac{\partial N_{c}^{(1)}}{\partial \zeta }\right) +\frac{\partial N_{c}^{(1)}}{\partial \tau }\nonumber \\&\quad -\frac{CN_{c}^{(0)}}{B^{(0)}\Omega _{c}}U_{ph}\left( 1-K_z^2\right) \frac{\partial ^3\Phi ^{(1)}}{\partial \zeta ^3}. \end{aligned}$$

Using Equations (16–26) we can find the following Korteweg de-Vries (KdV) equation for EAWs as

27 $$\begin{aligned} \frac{\partial \Psi (\zeta ,\tau )}{\partial \tau }+A\Psi (\zeta ,\tau )\frac{\partial \Psi (\zeta ,\tau )}{\partial \zeta } +B\frac{\partial ^3\Psi (\zeta ,\tau )}{\partial \zeta ^3}=0, \end{aligned}$$

where $A=\left( \frac{A_2}{A_1}\right)$ and $B=\left( \frac{A_3}{A_1}\right)$ corresponds to the nonlinearity and dispersion coefficients respectively. Where

$$\begin{aligned} A_1&=  \frac{2C_1 eN_{h}^{(0)}}{K_BT_h N_{c}^{(0)}K_z} \bigg (K_z U^{(0)}-U_{ph}\bigg ),\\ A_2&=  \frac{C_1 e^2N_{h}^{(0)}}{K_B^2T_h^2K_z(N_{c}^{(0)})^2} \Bigg \{\left( 3C_1 N_{h}^{(0)}+N_{c}^{(0)}\right) \left( K_z^2 (U^{(0)})^2-2K_z U_{ph} U^{(0)}+U_{ph}^2\right) -3 U_{Tc}^2 \left( N_{c}^{(0)}-C_1 N_{h}^{(0)}\right) \Bigg \},\\ A_3&=  \frac{1}{4K_z}\Bigg \{\frac{1}{B^{(0)} \Omega _{c} }\bigg \{4 C U_{ph} (1-K_z^2)(K_z U^{(0)}-U_{ph})\bigg \}\\&\quad +\frac{1}{\pi eN_{c}^{(0)}U_{Th}^2}\bigg \{-2 K_z U_{ph} U^{(0)} \left( 2 \pi m_e (1-K_z^2)(3 C_1 N_{h}^{(0)} U_{Tc}^2+N_{c}^{(0)} U_{Th}^2)-U_{Th}^2\right) \\&\quad +U_{ph}^2 \bigg (4 \pi m_e (1-K_z^2) \big (3 C_1 N_{h}^{(0)} U_{Th}^2+N_{c}^{(0)} U_{Th}^2\big )-U_{Th}^2\bigg )-K_z^2 U_{Th}^2 \big ((U^{(0)})^2-3 U_{Tc}^2\big )\bigg \}\Bigg \}, \end{aligned}$$

also $\Psi \left( =\Phi ^{(1)}/\Phi _0\right)$ with $\Phi _0\left( =10^{-11}statV\right) .$ For real values of all the parameters we have $A<0$ and $B>0$, give rise to the negative potential of the EAWs pulses. To obtain a localized stationary solitary waves solution moving to the right, we transform the independent variables $\zeta$ and $\tau$ to new moving coordinates ($\xi =\zeta -\mu _0\tau$), where $\mu _0$ is the speed of solitary waves in the new coordinate system. Also applying the vanishing conditions as $\Psi \rightarrow 0$, $d\Psi /d\xi \rightarrow 0$, $d^2\Psi /d\xi ^2\rightarrow 0$ at $\xi \rightarrow \pm \infty$, the localized solution of the equation (27) can be obtained as

28 $$\begin{aligned} \Psi (\xi )=\delta _0\sec h^2\bigg (\frac{\xi }{\Delta }\bigg ), \end{aligned}$$

where $$\delta _0\left( =\frac{3\mu _0}{A}\right) $$ and $$\Delta \left( =2\sqrt{\frac{B}{\mu _0}}\right) $$ represent the amplitude and spatial width of the EA solitons. The product $$\delta _0\Delta ^2\left( 
=12B/A\right) $$ giving the constant values independent of $$\mu _0$$ will suggest that the taller the amplitudes of the solitary waves will result in the faster and narrower the pulse shape accordance with the KdV theory. $$\Delta \left( =2\sqrt{\frac{B}{\mu _0}}\right) $$ suggests that increasing the solitary wave speed $$\mu _0$$ will increase the amplitudes but a decrease will occur in its width. The expression for the electric field can be calculated as

29 $$\begin{aligned} E=-\nabla \Psi =\frac{3\mu _0^{3/2}}{AB^{1/2}}\sec h^2\bigg (\frac{\xi }{\Delta }\bigg ) \tan h\bigg (\frac{\xi }{\Delta }\bigg )\bigg (K_x\hat{X}\quad K_y\hat{Y}\quad K_z\hat{Z}\bigg )^{-1}, \end{aligned}$$

where $\hat{X}$, $\hat{Y}$, and $\hat{Z}$ are the unit vectors along *X*, *Y* and *Z*-axis respectively.

## Modified double Laplace decomposition method (MDLDM)

This method is used here for the first to study the nonlinear evolution of solitary potential in nonextensive plasma. The modified double Laplace decomposition method has also extensively used to reduce the spatiotemporal solutions corresponds for the linear as well as nonlinear deferential equation^40–44^.

To discuss this method. consider the following non-linear problem of the form

30 $$\begin{aligned} L\phi (X,T)+R\phi (X,T)+N\phi (X,T)=f(X,T),~~~~~~~~\forall ~~~~ T\in R, \end{aligned}$$

where *L* is highest order linear operator, ($L=D^n(X,T)=\partial ^n\phi (X,T)/\partial X^n$), *N* is non-linear operator, and *R* is the operator contains the linear terms while *f*(*X*, *T*) is some external function.

Consider a function $\phi (X,T)$ for $X,T>0$ in $XT-plane$, the double Laplace transform of the function $\phi (X,T)$ is defined by^45^

$$\begin{aligned} L_XL_T\left[ \phi (X,T)\right] =\int _0^{\infty }e^{-S_1X}\left( \int _0^{\infty } e^{-S_2{T}}\,\phi (X,T)\,dT\ \right) dX, \end{aligned}$$

where $S_1$ and $S_2$ are complex numbers. The double Laplace transform for the partial derivatives of the function $\phi (X,T)$ can be represented as

31 $$\begin{aligned} L_XL_T\left\{ \frac{\partial ^n\,\phi (X,T))}{\partial X^n}\right\}&=  S_1^n\,\bar{\phi }(S_1,S_2)-\sum _{k=0}^{n-1}S_1^{n-1-k} L_T\left\{ \frac{\partial ^k{\phi (0,T)}}{\partial {X^k}} \right\} , \end{aligned}$$

and

32 $$\begin{aligned} L_XL_T\left\{ \frac{\partial ^m\phi (X,T))}{\partial T^m}\right\}&= S_1^m\bar{\phi }(S_1,S_2)-\sum \limits _{j=0}^{m-1}S_1^{m-1-j} L_{X}\Big \{ \frac{\partial ^j{\phi (X,0)}}{\partial {T^{j}}} \Big \}, \end{aligned}$$

where    $n,m=1,2,3,\cdots$. From the above definitions, we can deduce that

$$\begin{aligned} L_XL_T{\phi (X)\,\psi (T)}=\bar{\phi }(S_1)\bar{\Psi }(S_2)=L_X{\phi (X)}L_T{\Psi (T)}. \end{aligned}$$

  The inverse double laplace transform $L_X^{-1}L_T^{-1}\{\bar{\phi }(S_1,S_2)\}=\phi (X,T)$, is represented by a complex double integral formula

33 $$\begin{aligned} L_X^{-1}L_T^{-1}\Big \{\bar{\phi }(X,T)\Big \}&=  \frac{1}{2\pi \,i}\int \limits _{c-i\infty }^{c+i\infty }e^{S_{2} T}\left( \int \limits _{d-i\infty }^{d+i\infty }e^{S_{1} X} \bar{\phi }(S_1,S_2)\,dS_1\right) dS_2. \end{aligned}$$

  It should be noted that $\bar{\phi }\left( S_{1},S_{2}\right)$, is an analytic function $\forall$ $S_1$ and $S_2$ defined in the region by the inequalities *Re* $(S_1)\ge c$ and *Re* $(S_2)\ge d$, where $c, d \in R$ to be considered accordingly.

With the help of these definitions, apply double Laplace transform on both sides of Eq. (30) we obtained

34 $$\begin{aligned} \begin{aligned}{}&L_XL_T\{D^n\phi (X,T)\}+RL_XL_T\{\phi (X,T)\}+NL_XL_T\{\phi (X,T)\} =L_XL_T\{f(X,T)\}. \end{aligned} \end{aligned}$$

  Using the idea of double Laplace on nth-derivative, we obtained

35 $$\begin{aligned} \begin{aligned} L_XL_T\{\phi (X,T)\}&=G(S_1,S_2)-\frac{1}{S_2^n}RL_XL_T \{\phi (X,T)\}-\frac{1}{S_2^n}NL_XL_T\{\phi (X,T)\}, \end{aligned} \end{aligned}$$

where

36 $$\begin{aligned} G(S_1,S_2)=\frac{1}{S_2^n}\sum _{j=0}^{n-1}S_2^{n-1-j} L_X\left\{ \frac{\partial ^j{\phi (X,0)}}{\partial {X^j}} \right\} +\frac{1}{S_2^n} F(S_1,S_2). \end{aligned}$$

  Here we consider the series solution of the form

$$\begin{aligned} \phi (X,T)=\sum _{n=0}^\infty \phi _n(X,T), \end{aligned}$$

the non-linear terms are decomposed as

$$\begin{aligned} N\phi (X,T)=\sum _{i=0}^\infty A_n, \end{aligned}$$

where $A_n$ are called Adomian polynomials^46^ of $\phi ^{(0)},\phi ^{(1)},\phi ^{(2)}\cdots$, given by the following formula

37 $$\begin{aligned} A_n=\frac{1}{n!}\frac{d^n}{d\lambda ^n}\left[ \sum _{k=0}^n \lambda ^k\phi _k(X,T)\right] _{\lambda =0}. \end{aligned}$$

  Hence applying inverse double Laplace on both sides of Eq. (35) and with the help of Eq. (37) we obtain

$$\begin{aligned}\begin{aligned} \sum _{n=0}^\infty \phi _n(X,T)=L_X^{-1}L_T^{-1}G(S_1,S_2)-L_X^{-1}L_T^{-1}\left\{ \frac{1}{S_2^n}RL_XL_T \{\phi (X,T)\}\right\} -L_X^{-1}L_T^{-1}\left\{ \frac{1}{S_2^n}L_XL_T \sum _{n=0}^\infty A_n\right\} , \end{aligned} \end{aligned}$$

comparing the terms on both sides we get

$$\begin{aligned} \begin{aligned}{}&\phi ^{(0)}=L_X^{-1}L_T^{-1}[G(S_1,S_2),]\\ {}&\phi ^{(1)}=-L_X^{-1}L_T^{-1} \left\{ \frac{1}{S_2^n} L_XL_T[R\phi ^{(0)}]\right\} -L_X^{-1}L_T^{-1}\left\{ \frac{1}{S_2^n}L_XL_T[A_0]\right\} ,\\&\phi ^{(2)}=-L_X^{-1}L_T^{-1}\left\{ \frac{1}{S_2^n}L_XL_T[R\phi ^{(1)}]\right\} -L_X^{-1}L_T^{-1} \left\{ \frac{1}{S_2^n}L_XL_T[A_1]\right\} ,\\&\phi _3=-L_X^{-1}L_T^{-1}\left\{ \frac{1}{S_2^n}L_XL_T[R\phi ^{(2)}]\right\} -L_X^{-1}L_T^{-1} \left\{ \frac{1}{S_2^n}L_XL_T[A_2]\right\} ,\\&\vdots \\&\phi _{n+1}=-L_X^{-1}L_T^{-1}\left\{ \frac{1}{S_2^n} L_XL_T[R\phi ^{(n)}]\right\} -L_X^{-1}L_T^{-1} \left\{ \frac{1}{S_2^n}L_XL_T[A_n]\right\} , \end{aligned} \end{aligned}$$

the final solution can be obtain as

38 $$\begin{aligned} \phi (X,T)=\sum _{n=0}^\infty \phi ^{(n)}(X,T). \end{aligned}$$

## Applications of MDLDM

In this section, we consider our model (27) with $\Psi (\zeta ,\tau )=\phi (\xi ,\tau )$) and apply MDLDM as discussed in (“Modified double Laplace decomposition method (MDLDM)” section) to obtained an approximate solution to the problem. For this, we can write Eq. (27) in the following non-linear form

39 $$\begin{aligned} \phi _{\tau }(\xi ,\tau )+A\phi (\xi ,\tau )\phi _{\xi }(\xi ,\tau )+ B\phi _{\xi \xi \xi }(\xi ,\tau )=0,~~~~~~~~\forall ~~~~ \xi , \tau \in R, \end{aligned}$$

with initial condition

40 $$\begin{aligned} \phi (\xi ,0)=\delta _0\sec h^2\bigg (\frac{\xi }{\Delta }\bigg ), \end{aligned}$$

where $\delta _0\left( =\frac{3\mu _0}{A}\right)$ and $\Delta \left( =2\sqrt{\frac{B}{\mu _0}}\right)$ represent amplitude and spatial width of the EAWs. The variables in subscript of $\phi$ in Eq. (39) represent partial derivatives with respect to $\tau$ and $\xi$. Applying the definitions and techniques discussed in “Modified double Laplace decomposition method (MDLDM)” section on Eq. (39) as

41 $$\begin{aligned} \begin{aligned}{}&\phi ^{(0)}=L_\xi ^{-1}L_T^{-1}[G(S_1)],\\ {}&\phi ^{(1)}=-AL_\xi ^{-1}L_T^{-1} \left\{ \frac{1}{S_2} L_\xi L_T[A_0]\right\} -BL_\xi ^{-1}L_\tau ^{-1}\left\{ \frac{1}{S_2}L_\xi L_\tau [\phi _{\xi \xi \xi }^{(0)}]\right\} ,\\&\phi ^{(2)}=-AL_\xi ^{-1}L_\tau ^{-1}\left\{ \frac{1}{S_2}L_\xi L_\tau [A_1]\right\} -BL_\xi ^{-1}L_\tau ^{-1} \left\{ \frac{1}{S_2}L_\xi L_\tau [\phi _{\xi \xi \xi }^{(1)}]\right\} ,\\&\phi ^{(3)}=-AL_\xi ^{-1}L_\tau ^{-1}\left\{ \frac{1}{S_2}L_\xi L_\tau [A_2]\right\} -BL_\xi ^{-1}L_\tau ^{-1} \left\{ \frac{1}{S_2}L_\xi L_\tau [\phi _{\xi \xi \xi }^{(2)}]\right\} ,\\&\vdots \\&\phi ^{(n)}=-AL_\xi ^{-1}L_\tau ^{-1}\left\{ \frac{1}{S_2}L_\xi L_\tau [A_n]\right\} -BL_\xi ^{-1}L_\tau ^{-1} \left\{ \frac{1}{S_2}L_\xi L_\tau [\phi _{\xi \xi \xi }^{(n)}]\right\} , \end{aligned} \end{aligned}$$

where $G(S_1)$ and $A_0, A_1,A_2\cdots \cdots$ can be obtained by using Eqs. (36) and (37) as

42 $$\begin{aligned} G(S_1)&=  \delta _0\sec h^2\bigg (\frac{\xi }{\Delta }\bigg )=\phi (\xi ,0), \end{aligned}$$

43 $$\begin{aligned} A_0&=  \phi ^{(0)}\phi _{\xi }^{(0)}, A_1=\phi ^{(0)}\phi _{\xi }^{(1)}+\phi ^{(1)}\phi _{\xi }^{(0)}, A_2=\phi ^{(0)}\phi _{\xi }^{(2)}+\phi ^{(1)}\phi _{\xi }^{(1)}+\phi ^{(2)}\phi _{\xi }^{(0)}. \end{aligned}$$

Using Equations (42) and (43) in Eq. (41) we get the the following solution

$$\begin{aligned} \begin{aligned} \phi ^{(0)}&=\delta _0\sec h^2\left( \frac{\xi }{\Delta }\right) ,\\ \phi ^{(1)}&=\tau \left[ 8\delta _0\Delta ^{-3}B\sec h^2\left( \frac{\xi }{\Delta }\right) \tanh \left( \frac{\xi }{\Delta }\right) +2\delta _0\Delta ^{-3}\left( \delta _0\Delta ^2A-12B\right) \sec h^4\left( \frac{\xi }{\Delta }\right) \tanh \left( \frac{\xi }{\Delta }\right) \right] ,\\ \phi ^{(2)}&=\tau ^2 \delta _0\bigg [\left( -4\delta _0^2\Delta ^{-2}A^2+148\Delta ^{-4}AB-1208\Delta ^{-6}B^2 \right) \sec h^8(\frac{\xi }{\Delta })\\&\quad +\left( 3\delta _0^2\Delta ^{-2}A^2-136\delta _0 \Delta ^{-4}AB+1191\Delta ^{-6}B^2\right) \left( 2\sec h^6\left( \frac{\xi }{\Delta }\right) - \sec h^8\left( \frac{\xi }{\Delta }\right) \right) \\ {}&\quad +10\left( \delta _0\Delta ^{-4}AB-12\Delta ^{-6}B\right) \left( \sec h^4\left( \frac{\xi }{\Delta }\right) + \sec h^4\left( \frac{\xi }{\Delta }\right) \tanh ^4\left( \frac{\xi }{\Delta }\right) \right) \\&\quad +60\left( \delta _0\Delta ^{-4}AB-12\Delta ^{-6}B\right) \sec h^4\left( \frac{\xi }{\Delta }\right) \tanh ^2\left( \frac{\xi }{\Delta }\right) +\Delta ^{-6}B\sec h^2\left( \frac{\xi }{\Delta }\right) + 15\Delta ^{-6}B^2\sec h^2\left( \frac{\xi }{\Delta }\right) \tanh ^2\left( \frac{\xi }{\Delta }\right) \\&\quad +15\Delta ^{-6}B^2\sec h^2\left( \frac{\xi }{\Delta }\right) \tanh ^4\left( \frac{\xi }{\Delta }\right) +\Delta ^{-6}B^2\sec h^2\left( \frac{\xi }{\Delta }\right) \tanh ^6\left( \frac{\xi }{\Delta }\right) \bigg ], \end{aligned} \end{aligned}$$

similarly, the other terms can be calculated in the same manner. The final solutions can be obtained

44 $$\begin{aligned} \phi (\xi ,\tau )=\sum _{n=0}^2 \phi ^{(n)}(\xi ,\tau ). \end{aligned}$$

**Table 1:** Comparison of localized solution with approximate solution for $\mu _0=1$,and $\bar{\tau } (=\tau \omega _0)=0.1$.

$\bar{\xi }$	$\bar{\zeta }$=$\bar{\xi }-\mu _0\bar{\tau }$	Localized solution	MDLDM Solution	Absolute Error
− 3.0	− 3.1	− 2.2648$\times 10^{-13}$	− 5.0807$\times 10^{-13}$	2.816$\times 10^{-13}$
− 2.5	− 2.6	− 3.132$\times 10^{-11}$	− 8.2781$\times 10^{-11}$	5.146$\times 10^{-11}$
− 2.0	− 2.1	− 4.3314$\times 10^{-09}$	− 1.1595$\times 10^{-08}$	7.2638$\times 10^{-09}$
− 1.5	− 1.6	− 5.9901$\times 10^{-07}$	− 1.6053$\times 10^{-06}$	1.0063$\times 10^{-06}$
$\cdots$	$\cdots$	$\cdots$	$\cdots$	$\cdots$
$\cdots$	$\cdots$	$\cdots$	$\cdots$	$\cdots$
$\cdots$	$\cdots$	$\cdots$	$\cdots$	$\cdots$
$\cdots$	$\cdots$	$\cdots$	$\cdots$	$\cdots$
$\cdots$	$\cdots$	$\cdots$	$\cdots$	$\cdots$
1.5	1.4	− 4.3028$\times 10^{-06}$	− 1.6056$\times 10^{-06}$	2.6972$\times 10^{-06}$
2.0	1.9	− 3.1114$\times 10^{-08}$	− 1.1623$\times 10^{-08}$	1.9491$\times 10^{-08}$
2.5	2.4	− 2.2498$\times 10^{-10}$	− 8.5107$\times 10^{-11}$	1.3988$\times 10^{-11}$
3.0	2.9	− 1.6268$\times 10^{-12}$	− 7.0592$\times 10^{-13}$	9.2093$\times 10^{-13}$

**Table 2:** Comparison of localized solution with approximate solution for $\mu _0=1$,and $\bar{\tau }=0.01$.

$\bar{\xi }$	$\bar{\zeta }$=$\bar{\xi }-\mu _0\bar{\tau }$	Localized solution	MDLDM Solution	Absolute Error
− 3.0	− 3.01	− 5.5001$\times 10^{-13}$	− 5.971$\times 10^{-13}$	4.7095$\times 10^{-14}$
− 2.5	− 2.51	− 7.6063$\times 10^{-11}$	− 8.3828$\times 10^{-11}$	7.7646$\times 10^{-12}$
− 2.0	− 2.01	− 1.0519$\times 10^{-08}$	− 1.1608$\times 10^{-08}$	1.0885$\times 10^{-09}$
− 1.5	− 1.51	− 1.4547$\times 10^{-06}$	− 1.6054$\times 10^{-06}$	1.5071$\times 10^{-07}$
$\cdots$	$\cdots$	$\cdots$	$\cdots$	$\cdots$
$\cdots$	$\cdots$	$\cdots$	$\cdots$	$\cdots$
$\cdots$	$\cdots$	$\cdots$	$\cdots$	$\cdots$
$\cdots$	$\cdots$	$\cdots$	$\cdots$	$\cdots$
$\cdots$	$\cdots$	$\cdots$	$\cdots$	$\cdots$
1.5	1.49	− 1.7718$\times 10^{-06}$	− 1.6055$\times 10^{-06}$	1.6633$\times 10^{-07}$
2.0	1.99	− 1.2812$\times 10^{-08}$	− 1.161$\times 10^{-08}$	1.2014$\times 10^{-09}$
2.5	2.49	− 9.2641$\times 10^{-11}$	− 8.406$\times 10^{-11}$	8.5812$\times 10^{-12}$
3.0	2.99	− 6.6989$\times 10^{-13}$	− 6.1689$\times 10^{-13}$	5.2999$\times 10^{-14}$

## Results and discussions

For numerical illustrations, we choose an electron-ion (EI) plasma that comprises for superthermal electrons, and cold electrons as well as positively charged ions. The number density and magnetic field for the plasma ranges $N^{(0)}=10^{18}\,\mathrm{cm}^{-3}-10^{20}\,\mathrm{cm}^{-3}$, and $B^{(0)}=10^{5}G-10^{7}G$ respectively. Moreover, the temperature for superthermal (cold) electrons are taken as $T_h=10^4K(10^3K)$ respectively. Such plasma has relevance to the Earth magnetosphere, the ionosphere, the laboratory facilities. For the purpose in numerical illustrations, we have normalized the phase speed $(U_{ph})$, the non-linearity coefficient (A), the dispersion coefficient (B), the wave amplitude $(\Psi ,\phi )$ with $U_{ph0}=10^7\,\mathrm{cm}\,\mathrm{s}^{-1}$, $A^{(0)}=10^{11}\,\mathrm{cm}(statVs)^{-1}$, $\phi _0=10^{-10}statV$, respectively.

The important results of our study are presented in the following discussion.

**Figure 2:**
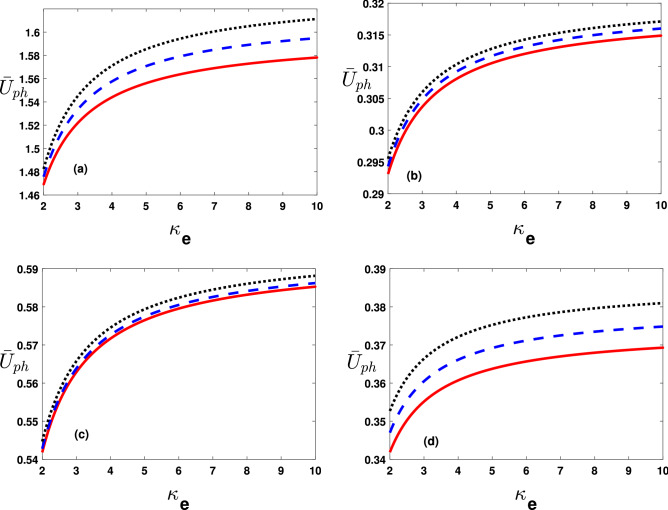
Phase speed ($\bar{U}_{ph}$=$U_{ph}/U_{ph_0}$ with $U_{ph_0}=10^7\,\mathrm{cm}\,\mathrm{s}^{-1}$) varying with $\kappa _e$ from 2 to 10 by changing (**a**) hot electron temperature values as $T_h=10^3K$ (solid curve), $1.1\times 10^3K$ (dashed curve) and $1.2\times 10^3K$ (dotted curve), (**b**) cold electron temperature values as $T_c=10^3K$ (solid curve), $1.01\times 10^3K$ (dashed curve) and $1.02\times 10^3 K$ (dotted curve), (**c**) $U^{(0)}$ values as $U^{(0)}=10^5\,\mathrm{cm}\,\mathrm{s}^{-1}$ (solid curve), $1.2\times 10^5\,\mathrm{cm}\,\mathrm{s}^{-1}$ (dashed curve) and $1.6\times 10^5\,\mathrm{cm}\,\mathrm{s}^{-1}$(dotted curve) and (**d**) $\bar{K}_z$ values as $\bar{K}_z(=K_z/K_0)=0.6$ (solid curve), 0.609 (dashed curve) and 0.619 (dotted curve).

Figure 2a depicts the dimensionless phase speed $\bar{U}_{ph}(=U_{ph}/U_{ph0}$, verses the superthermal index $(\kappa _e)$, for the electron-acoustic (EA) solitary pulse at $T_h=10^3K$(solid curve), $1.1\times 10^3K$(dashed curve),$1.2\times 10^3K$(dotted curve). It reveals that the thermal correction of superthermal electrons decreases $\bar{U}_{ph}$. We have displayed $\bar{U}_{ph}$ against $\kappa _e$ with variations in Fig. 2b temperature due to the dynamical electrons $T_c=10^3K$(solid curve), $1.01\times 10^3K$(dashed curve),$1.02\times 10^3K$(dotted curve). See the degree enhancement in $T_c$ gives rise to $\bar{U}_{ph}$. In a similar fashion, Fig. 2c,d, illustrate $\bar{U}_{ph}$ verses $\kappa _e$ with variation in the electron streaming speed $(U^{(0)})$, and obliquity parameter $(\bar{K}_z)$. It infer that both the streaming electrons and the obliqueness $(\bar{K}_z)$ enhances the phase speed $\bar{U}_{ph}$.

**Figure 3:**
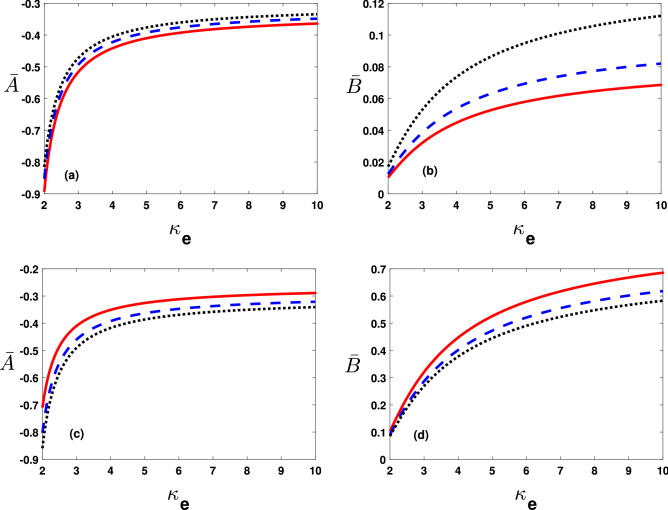
The coefficients $\bar{A}$(=$A/A^{(0)}$ with $A^{(0)}=10^{11}\,\mathrm{cm}(statVs)^{-1}$) and $\bar{B}$ (=$B/B_0$ with $B_0=10^{-8}\,\mathrm{cm}^3\,s^{-1}$) varying with $\kappa _e$ from 2 to 10 (**a**) by changing hot electrons temperature values for $\bar{A}$ as $T_h=10^3K$ (solid curve), $1.1\times 10^3K$ (dashed curve) and $1.3\times 10^3K$(dotted curve), (**b**) hot electrons temperature values for $\bar{B}$ as $T_h=10^3K$ (solid curve), $1.05\times 10^3K$ (dashed curve) and $1.1\times 10^3K$(dotted curve), (**c**) by changing cold electrons temperature values for $\bar{A}$ as $T_c=10^3$ (solid curve), $1.3\times 10^3$ (dashed curve) and $1.5\times 10^3K$ (dotted curve) and (**d**) by changing cold electrons temperature values $\bar{B}$ as $T_c=10^3K$ (solid curve), $1.3\times 10^3K$ (dashed curve) and $1.5\times 10^3K$ (dotted curve).

The lower and upper panels in Fig. 3a,b illustrate the dimensionless non linearity $\bar{A}(=A/A^{(0)})$ and dispersion $\bar{B}(=B/B_0)$ coefficients, respectively with variations in thermal effects of hot (cold) electrons, i.e., $T_h\left( T_c\right)$. Importantly note, thermal effect spread out the super-thermal electrons that in turn rises $\bar{A}$ and $\bar{B}$. Moreover, opposite trend notices for $\bar{A}$ and $\bar{B}$ with enhancement in $T_c$ as shown in Fig. 3c,d.

**Figure 4:**
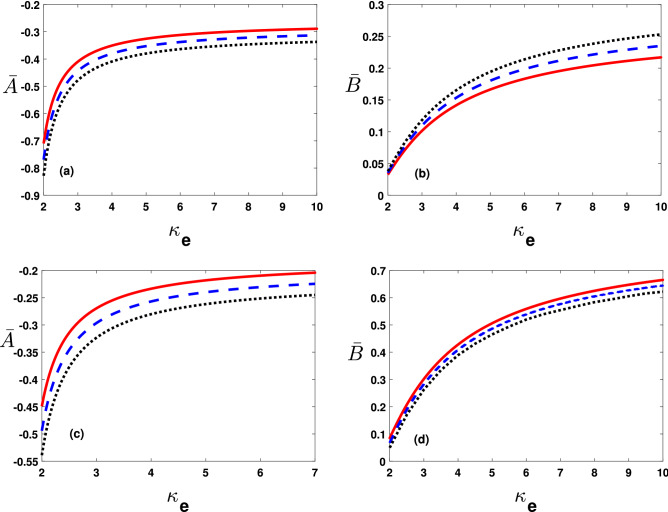
The coefficients $\bar{A}$(=$A/A^{(0)}$ with $A^{(0)}=10^{11}\,\mathrm{cm}(statVs)^{-1}$) and $\bar{B}$ (=$B/B_0$ with $B_0=10^{-8}\,\mathrm{cm}^3\,s^{-1}$) varying with $\kappa _e$ from 2 to 10 by changing (**a**,**b**) $\bar{K_z}$ values as $\bar{K}_z=0.6$ (solid curve), 0.7 (dashed curve) and 0.8 (dotted curve) and (**c,d**) $\bar{A}$ and $\bar{B}$ varying against $\kappa _e$ for different values of $U^{(0)}$=$5\times 10^{12}\,\mathrm{cm}\,\mathrm{s}^{-1}$ (solid curve), $10\times 10^{12}\,\mathrm{cm}\,\mathrm{s}^{-1}$ (dashed curve) and $15\times 10^{12}\,\mathrm{cm}\,\mathrm{s}^{-1}$ (dotted curve).

For the impact of relevant plasma parameters on the nonlinear steepening and dispersions effects, we have plotted in Fig. 4a–d coefficients $\bar{A}$ and $\bar{B}$ at different values of $\bar{K_z}$ and $U^{(0)}$ respectively. Obviously, $\bar{K_z}$ and $U^{(0)}$ reduces coefficient $\bar{A}$ and $\bar{B}$. Thus it reveals that oblique propagation of EA excitations suffer reduction in the nonlinear pulse steepening and dispersion.

**Figure 5:**
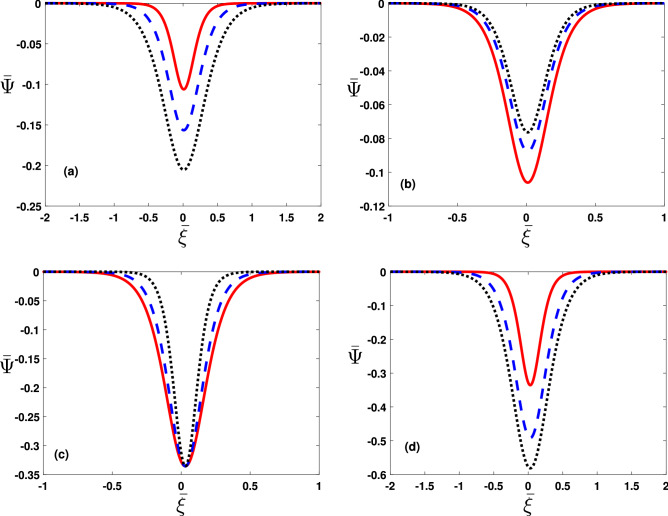
The approximate solution ($\bar{\Psi }=\phi /\phi _0$ with $\phi _0=10^{-11}statV$ given in Eq. (44) is taken against the spatial variable $\bar{\xi } (=\xi K_0)$ by changing (**a**) hot electrons temperature values as $T_h=10^3K$ (solid curve), $1.1\times 10^3K$ (dashed curve) and $1.2\times 10^3K$ (dotted curve), (**b**) cold electrons temperature values as $T_c=10^3K$ (solid curve), $1.5\times 10^3K$ (dashed curve) and $2.5\times 10^3K$ (dotted curve), (**c**) $U^{(0)}$ values as $2\times 10^{12}\,\mathrm{cm}\,\mathrm{s}^{-1}$ (solid curve), $10\times 10^{12}\,\mathrm{cm}\,\mathrm{s}^{-1}$ (dashed curve) and $20\times 10^{12}\,\mathrm{cm}\,\mathrm{s}^{-1}$ (dotted curve) and (**d**) changing $\kappa _e$ values as 2 (solid curve), 3 (dashed curve) and 5 (dotted curve).

To show the impact of electronic temperature, we have given the wave solution (27) for pulse-shaped soliton against the spatial variable $\bar{\xi }$ (see Fig. 5a,b). Recall that $T_h(T_c)$ decreases(increases) coefficients $\bar{A}$ and $\bar{B}$, and therefore rises (reduces) the pulse amplitude and spatial extension for solitary potentials. Likewise, the streaming speed $(U^{(0)})$ and the superthermality index ($\kappa _e$) also impact the wave profiles as illustrated in Fig. 5c,d.

**Figure 6:**
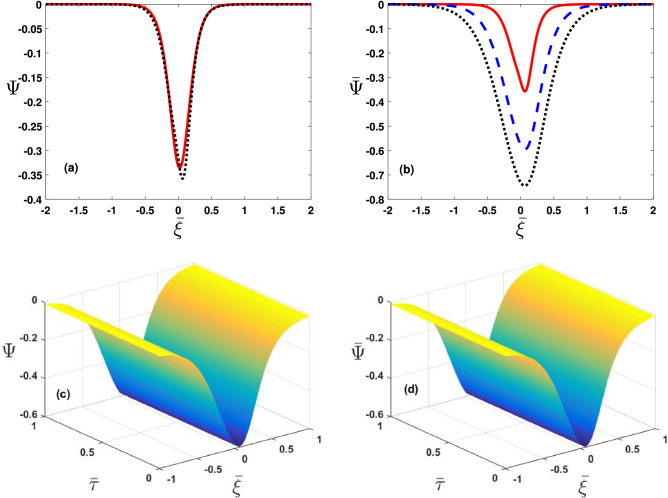
(**a**) Comparison of localized solution (28) (red curve) vs approximate solution (44)(dotted curve), (**b**) effect of $\kappa _e$=2 (solid curve), $\kappa _e$=3 (dashed curve) and $\kappa _e$=5 (dotted curve) on the MDLDM-solution. Similarly (**c**) 3D surface plot for localized solution and (**d**) 3D surface plot for approximate solution.

In Fig. 6a we compare our localized solution (27) with the series solution (44) obtained by the MDLDM method. Obviously, at $\bar{\tau }>0$ the series MDLDM solution admits spatial deviation with amplification in pulse amplitude. The MDLDM solution in Fig. 6b at different values of $\kappa _e$ shows that variation in superthermality index significantly modifies the EA soliton. The 3D surface plots for both the localized and approximate solution have been shown in Fig. 6c,d.

**Figure 7:**
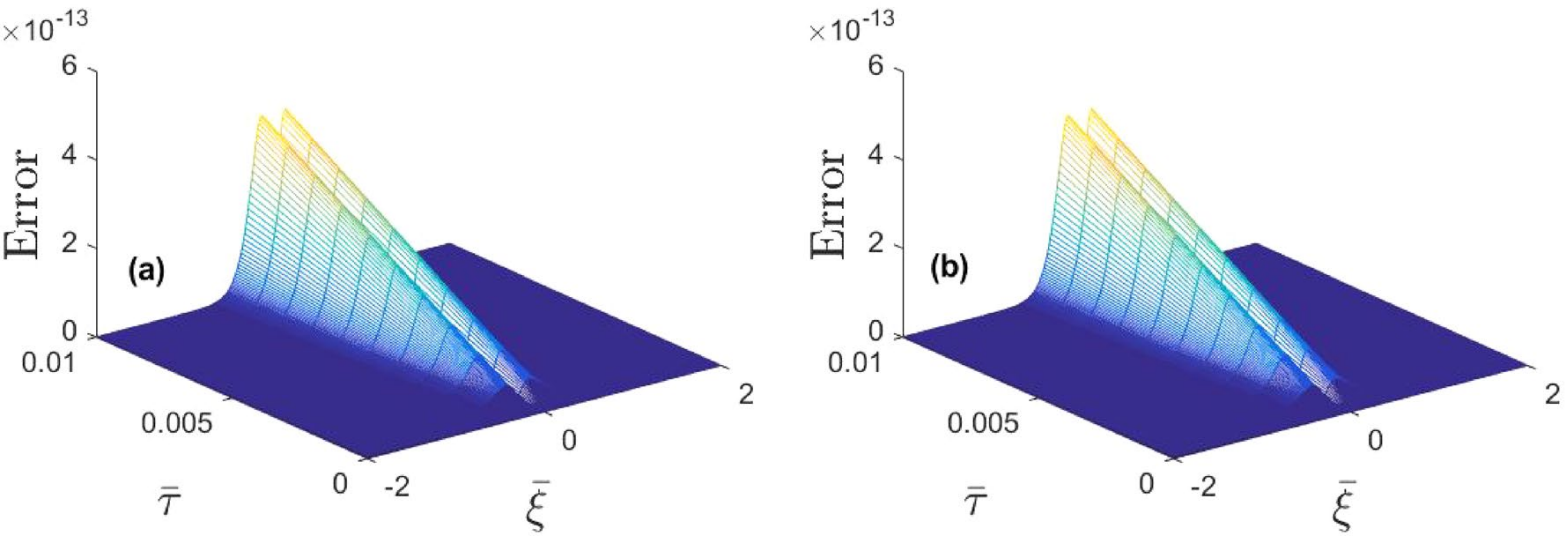
(**a**) 3D error plot for Table 1and (**b**) 3D error plot for Table 2.

**Figure 8:**
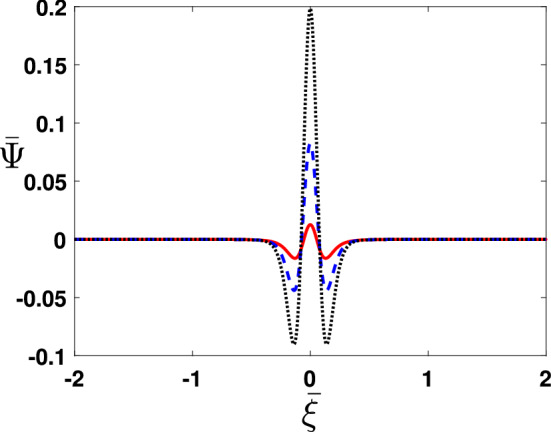
The approximate (MDLDM) solution is plotted for different values of $\bar{\tau }$=0.1 (solid curve), $\bar{\tau }$=0.2 (dashed curve) and $\bar{\tau }$=0.3 (dotted curve).

The MDLDM is an excellent tool to calculate analytical solutions of non-integrable system more accurately. The obtained results indicates that it is an effective tool for solving the KdV equation. One can see that the MDLDM solution satisfies the precise solution in Table 1 at $\bar{\tau } =0.1$ and Table 2 at $\bar{\tau }=0.01$, as well as in Fig. 7a,b. From tables, it is observed that the absolute error between the localized and approximate solution is reducing and approaching to zero by taking larger values for the spatial variable ($-3\le \bar{\xi }\ge 3$) with the temporal variable $\bar{\tau }=0.1$ and 0.01 respectively. As we know that, the KdV equation permits solitary wave solutions in plasma, and because MDLDM is a good and simple tool for studying the KdV equation, we may use this approach to numerically examine the localized behavior of solitons. It is also worth noting that MDLDM’s findings are quite near to those of precise solutions. We may use MDLDM for such non-linear equations to compare our results and eliminate mistakes in handling such equations because it is a simple approach.

Finally, Fig. 8 illustrates the MDLDM solution with variation in temporal variable as $\bar{\tau }=0.1$ (solid curve), 0.2 (dashed curve), and 0.3 (dotted curve), See enhancement in $\bar{\tau }$ oscillates the pulse shaped soliton and involves into subsequent deformation. It is to mention for rigor that MDLDM solution for KdV equation admit instability and growth of pulse shaped soliton that cannot be noticed in analytical techniques. The results further show two pulse profile of the approximate solution (MDLDM) when the time is increasing from 0.1 to 0.3 respectively. The time dependent solutions exhibits oscillation and deviating the stationary solution.

## Conclusion

We have examined the linear and nonlinear propagation characteristics of electron acoustics excitations in a nonextensive magnetoplasma. The latter contains cold dynamical electrons as well as super thermal electrons with positively charged stationary ions. It has been noticed that magnetosphere, ionospheres as well as laboratory scenarios are few among the possible outlets for the plasma conditions. For the linear stability analysis, we have deducted a fourth order linear dispersion relation admitting a positive imaginary root that corresponds to growth rate. We have shown the plasma streaming effect and the magnetic field strength give rise to instability growth. We have modelled a KdV equation for super thermal plasmas that is dependent on plasma characteristics such as hot electron temperature $T_h$, cold electron temperature $T_c$, shear flow speed $U^{(0)}$ direction cosine $\bar{K_z}$, phase speed $\bar{U}_{ph}$, and magnetic field $B^{(0)}$. The KdV equation is derived using a reductive perturbation approach for nonlinear analysis, and the solutions are quantitatively studied. The current findings are critical for comprehending nonlinear EA wave’s excitations in the presence of a magnetic field. The approximate solution of the model equation is calculated using MDLDM. The analytical and numerical results are compared where good agreement is obtained. It should be noted that the solution obtained by MDLDM for the governing model admit instability and growth of the pulse shaped solitons that cannot be noticed in other analytical techniques.

## Data Availability

The data regarding this work is available within the manuscript.
